# Organochlorine Pesticide Levels and Risk of Parkinson's Disease in North Indian Population

**DOI:** 10.1155/2013/371034

**Published:** 2013-07-08

**Authors:** Neelam Chhillar, Neeraj Kumar Singh, B. D. Banerjee, Kiran Bala, Md Mustafa, Deepika Sharma, Mitrabasu Chhillar

**Affiliations:** ^1^Department of Neurochemistry, Institute of Human Behaviour and Allied Sciences, Dilshad Garden, Delhi 110095, India; ^2^Environmental Biochemistry Laboratory, Department of Biochemistry, UCMS & G.T.B. Hospital (University of Delhi), Dilshad Garden, Delhi 110095, India; ^3^Department of Neurology, Institute of Human Behaviour and Allied Sciences, Dilshad Garden, Delhi 110095, India; ^4^Health Centre, Institute of Nuclear Medicine and Allied Sciences, DRDO, Timarpur, Delhi 110054, India

## Abstract

The cause of Parkinson's disease (PD) remains elusive, but environmental chemical exposures have been postulated to be involved in the etiology of PD. We examined the association between the persistent organochlorine pesticides (OCPs) and PD in the North Indian population. This case control study included 70 PD and 75 control subjects in the age group of 50 to 85 years. Blood samples were collected and high-purity grade hexane and acetone (2 : 1 ratio) were used for extraction of organochlorine residues. OCPs (hexachlorocyclohexane (HCH), aldrin, dieldrin, endosulfan, pp′-Dichlorodiphenyldichloroethylene (pp′-DDE), op′-DDE, pp′- Dichlorodiphenyltrichloroethane (pp′-DDT), op′-DDT, pp′-dichlorodiphenyldichloroethane (pp′-DDD) and op′-DDD) were quantitatively estimated by using gas chromatography. The most frequently detected OCP was dieldrin, which was present in 9.3% of control and 61.4% of PD. The strongest predictor was *β*-hexachlorocyclohexane (*β*-HCH), which reported an odds ratio of 2.566, indicating that for every additional one unit of *β*-HCH, patients had 2.566 times more chances of presence of PD. This study indicates that increased level of *β*-HCH and dieldrin may be associated with the risk of PD.

## 1. Introduction 

Neurodegenerative diseases form a subset of pathologies that are characterized by a progressive loss of neurons paralleled by the emergence of misfolded proteins in various cell types, the significance of which is still being debated [1]. Parkinson's disease (PD) is the second neurodegenerative disorder in importance after Alzheimer's disease, in which the movement-regulating cells of the brain get disabled, leading to tremors, slowed movement, balance problems, speech, and behaviour changes. The cause of PD remains elusive, but environmental chemical like pesticide exposures have been postulated to be involved in the etiology of PD [2, 3]. 

Worldwide, more than 25 million tons of pesticides are used every year, and 99% of these pesticides are being released aimlessly into the environment [4]. In India, pesticides are frequently used for agriculture development and protection/control of diseases like malaria, filariasis, dengue, Japanese encephalitis, cholera, and so forth. Synthetic organic pesticides are used to control weeds, insects, and other organisms in agricultural and nonagricultural settings. India is one of the few remaining countries still engaged in the large-scale manufacture, use, and export of some organochlorine pesticides (OCPs) (such as p,p′-dichlorodiphenyltrichloroethane (DDT), hexachlorocyclohexane (HCH), and pentachlorophenol) for agriculture and vector control. Despite this, few data are available on OCP levels in the urban atmosphere of India [5]. OCPs are persistent pesticides and concentrated up in the food chain. They can be detected in the diet including drinking water. Many OCPs induce nigrostriatal dopaminergic neurotoxicity through the generation of oxidative stress and mitochondrial dysfunction [6]. Concerns in regard to developmental neurotoxicity due to pesticides have been fuelled by recent epidemiologic observations that persons exposed prenatally or during early postnatal life suffer from various neurological deficits [7–9]. However, the exact pathways leading to pesticide-induced neurodegeneration remain elusive. The aim of this study was to examine the association between the persistent OCPs and PD in the North Indian population. 

## 2. Materials and Methods 

This case control study included 145 subjects in the age group of 50 to 85 years, during the period from February 2010 to January 2012, attending the outpatients department of neurology of a tertiary care hospital in New Delhi, India. Out of these, 70 subjects diagnosed with PD were enrolled in the study group. PD was defined by United Kingdom Parkinson's Disease Society Brain Bank Criteria [10]. Additional inclusion criteria were a score of >23 on Mini-Mental Status Examination (MMSE) and a Clinical Dementia Rating (CDR) score of <0.5 for PD. Control group (75 subjects) is comprised of age-matched healthy volunteers. Subjects (case and control) were excluded if there was a lack of consent to participate in the study, history of cerebral stroke, epilepsy, head trauma, and other concomitant disease potentially associated with PD and familial history of any kind of cognitive/behavioral abnormality. Routine laboratory tests were performed to rule out nutritional deficiency and metabolic abnormalities. The study was approved by the Institutional Ethical Committee. 

Blood samples were collected using standard venepuncture technique and stored at −80°C until analysis. Extraction of OCP residues was done by using HPLC- grade hexane and acetone (2 : 1) according to method of Singh et al. [11]. Hexane (6 mL) and acetone (3 mL) were added and the contents were shaken at room temperature for 30 minutes in a mechanical shaker. The clear top layer of hexane was collected in a clean test tube. The remaining portion was again extracted twice using the same process and the hexane fractions were added to the previous solvent fractions. Cleanup was done by USEPA method by column chromatography as described by Singh et al. [11]. Florisil was activated at 130°C overnight and cooled in a dessicator before use. 1 g of florisil was packed in the 20 cm length and 12 mm ID glass chromatographic column, anhydrous sodium sulfate was added to the top of the florisil column (0.5 cm), and the column was preeluted with hexane and discarded. The extract was transferred to the column and eluted with hexane (10 mL). Elute was collected in a 100 mL beaker and hexane was evaporated to concentrate the sample followed by analysis of sample by gas chromatography. 

The pesticides assayed were *α*-HCH, *β*-HCH, *γ*-HCH, aldrin, Dieldrin, *α*-endosulfan, *β*-endosulfan, *pp*′-DDE, p,p′-*pp*′-DDT, and *op*′ DDT. The samples were analysed on Perkin Elmer Gas Chromatography equipped with 63Ni electron capture detector under standard operating conditions [11]. For quantitative analysis of organochlorine residues in each sample, the peak area of each residue was compared with that obtained from a chromatogram of a mixed OCPs standard (Supelco, Sigma-Aldrich) of known concentration. The detection limit of the detector was <0.05 pg per chloroethylene with nitrogen as a carrier gas. The detection limit of the method was 4 pg/mL for each OCP. For quality control process, five blood samples in triplicate were spiked with a mixed standard of OCPs at 5 and 25 ng/mL. The average recoveries of fortified samples exceeded 95%. The case and control samples were run in the same analytical batches, and to maintain accuracy, a quality check sample was run always with each set of samples for pesticide analysis. 

## 3. Statistical Analysis

The statistical analysis was carried out by using SPSS version 20. *χ* ^2^ was performed for analysis of categorical variables, including sex and pesticide detection with the presence or absence of Parkinson's disease. The data was analysed for normality using Kolmogorov Smirnov and Shapiro-Wilk tests. OCPs were expressed as detected in patients (%), detectable range and 25th, 50th, and 75th percentile. Nonparametric test of significance (Mann Whitney Tests) was used to compare pesticide levels in two groups. Odds ratios (ORs) with 95% confidence intervals (CIs) were estimated using logistic regression analysis, with *β*-HCH, Dieldrin, *pp*′-DDE, age, and sex, as predictor variables for PD versus control status. Before putting the variables for logistic regression, all assumptions of multicollinearity and presence of outliers were tested by collinearity diagnostics and inspection of residuals respectively. Values of tolerance and variance inflation factor (VIF) were calculated to exclude multicollinearity. Variables with tolerance values of less than 0.1 and/or VIF values of more than 10 were excluded from the model. To evaluate the fit of the model to the data, Omnibus tests of model coefficients and computation of residuals using a Hosmer-Lemeshow test were performed. Amount of variation on dependent variable was calculated using Cox and Snell *R* square and Nagelkerke *R* square values. Variables with *P* value < 0.05 at Wald test were considered contributing significantly to the predictive ability of the model. 

## 4. Results 

**Figure 1:**
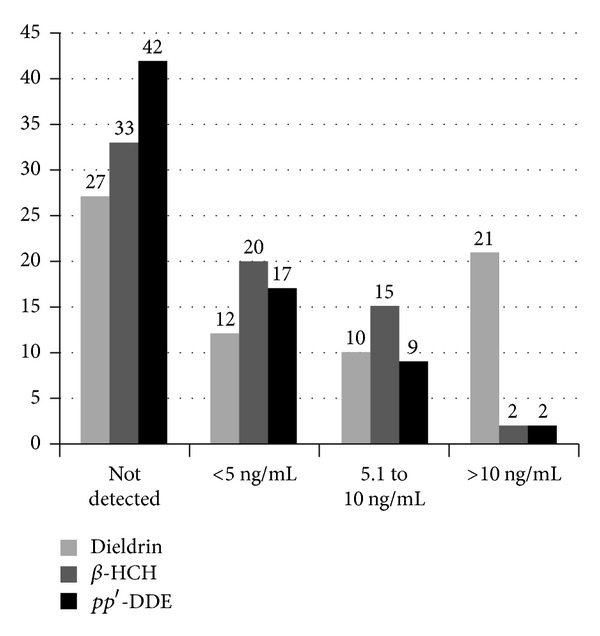
Distribution of different concentrations of organochlorine pesticide levels in Parkinson's disease (PD).

There was no significant difference in both the groups in terms of sex (*χ* ^2^ = 1.161; *P* = 0.281). A Mann-Whitney *U* test revealed no significant difference in the age of PD subjects (Md = 67.93, *n* = 70) and controls (Md *= *77.73, *n* = 75), *U* = 2270, *z* = −1.408, *P* = 1.59, *r* = 0.117. Distribution of OCP levels is listed in Table 1. OCPs were present in variable proportions in PD and controls but for statistical analysis, we have taken those OCPs which are present in ≥40% subjects of PD. *β*-HCH, Dieldrin, and *pp*′-DDE were present in ≥40% of PD subjects (Table 1). In Table 2 levels of *β*-HCH, Dieldrin, and *pp*′-DDE were observed to be significantly high in the PD subjects as compared to controls (*P* = 0.000, *P* = 0.000, and *P* = 0.008, resp.). Further we have divided these three OCP levels (*β*-HCH, Dieldrin, and *pp*′-DDE) into four different ranges of concentration (i.e., not detected, less than 5 ng/mL, 5.1 to 10 ng/mL, and greater than 10 ng/mL) as shown in Figure 1. 

Tolerance and VIF values in Table 3 indicate that there was no multicollinearity between independent variables of our model. Omnibus test of model coefficients showed that the full model containing all predictors was statistically significant, *χ* ^2^(5, *N* = 145) = 117.266, *P* < 0.00, indicating that the model was able to distinguish between controls and those suffering from PD. Further, Hosmer and Lemeshow test also supports our model (*χ* ^2^ = 5.3, *P* = 0.724) indicating an excellent fit. The model as a whole explained between 55.5% (Cox and Snell *R* square) and 74.0% (Nagelkerke *R* squared) of the variance in disease status, and correctly classified 88.3% of cases, when only 57.1% of cases could be classified without entering our independent variables in our model. 

**Figure 2:**
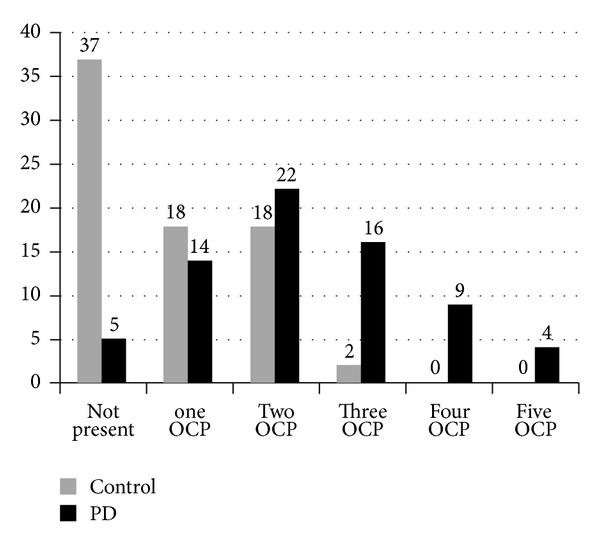
Number of organochlorine pesticide (OCP) present in Parkinson's Disease (PD) and control.

As shown in Table 3, only two of the independent variables made a unique statistically significant contribution to the model (*β*-HCH and Dieldrin levels). The strongest predictor was *β*-HCH, which reported an odds ratio of 2.566 (CI95% = 1.683, 3.912), indicating that, for every additional one unit of increase in *β*-HCH, subjects had 2.566 times more chances of developing PD.  Figure 2 shows that up to five OCPs were present in PD cases, and the maximum number of pesticides present in controls was three. *χ* ^2^ tests between two groups (pesticides present = 1) revealed significant difference (*χ* ^2^ = 49.5, df = 5, *P* = 0.00). 

## 5. Discussion 

Evidence tying pesticide use and PD have surfaced from all over the world although the identification of specific compounds has been more complex. Koller et al. [12] found that rural living and drinking well water were identified as risk factors for PD, which led to suspicion that pesticide exposure may increase the risk for PD. OCPs have been the most concerned class of pesticides with regard to the development of PD [13]. Recently studies have demonstrated that organochlorine pesticides are neurotoxic and can damage the dopamine system through generation of oxidative stress, proteasomal dysfunction, disruption of mitochondrial function, and increased alpha-synuclein levels and aggregation, all of which are associated with PD [13–15]. 

In the present study we found a significantly increased level of OCPs, mainly *β*-HCH, dieldrin, and p,p-DDE in subjects with PD as compared to controls, which is in accordance with the previous studies [16–19]. 

Chakraborty et al. [5] conducted passive and active air sampling in 7 metropolitan cities of India and found that the concentration of HCHs appears to be the highest reported across the globe. Hexachlorocyclohexane (HCH) is a synthetic chemical consisting of eight isomers. Only four of these isomers, *α*-HCH, *β*-HCH, *γ*-HCH, and *δ*-HCH, are of commercial significance. Among the HCH isomers, *β*-HCH is most slowly excreted from the body. In humans, the concentration of *β*-HCH in adipose tissue is typically higher than other HCH isomers [20]. In the present study, elevated serum levels of *β*-HCH have been found, which is in accordance with the recent study done by Richardson et al. [17]. Li et al. [21] estimated global usage of *β*-HCH from 1945 and 2000 at 8,50,000 tons, of which 2,30,000 tons were emitted into the atmosphere over the same period. Recently Richardson et al. [22] reported that the levels of *β*-HCH have decreased significantly between 2001 and 2008, but the association with PD was still significant and suggested that elevated levels of serum *β*-HCH in people over 60 years of age might serve as a useful predictor of PD development. Increased *β*-HCH levels have been linked with older age, rural environment livelihood, and male gender in humans, and these are identified as risk factors for PD. The presence of detectable *β*-HCH levels in control group holds the fact that other factors, like lifestyle or genetic polymorphism, may act together with *β*-HCH to increase the risk of development of PD. There may be a possible role of enzymes that regulate the xenobiotic transport and metabolism [23]. In the present study we also found that presence of *β*-HCH in blood will increase the risk of PD 2.5 times that is double of what Faroe Islands study showed (OR = 1.44), while Richardson et al. [17] showed the OR of 4.39. 

Recently some studies concluded that the Dieldrin is among the more likely candidate to contribute to the development of PD [14, 17]. In this present study, 21% of PD subjects show >10 ng/mL concentration of Dieldrin, pointing towards the emergent role of it in risk of PD, as other two pesticides (*β*-HCH and *pp*′-DDE) were found in only two cases each. We also found that the dieldrin also contributes to risk of PD as the OR calculated was 2.0 in PD subjects. Dieldrin has possibly the best documented toxicity on dopaminergic cells which includes the generation of oxygen radicals, aggregation and fibrillation of *α*-synuclein, disruption of the ubiquitin-proteasome system and the mitochondrial membrane potential, induction of dopamine release leading to intracellular dopamine depletion, and activation of caspases [14, 24, 25]. One study showed that mice exposed to Dieldrin depict similar pathologic effects that have been seen in PD, such as increased oxidative stress, increased *α*-synuclein expression, and altered dopamine homeostasis [14]. Few small studies were pointing toward the association of Dieldrin exposure with Parkinson's disease [19, 20]. The cell culture models of PD showed that dopaminergic neurons are more sensitive to Dieldrin-induced neurotoxicity [19, 24]. 

Serum *pp*′-DDE levels in present study were also significantly high in PD subjects as compared to controls. It is found to be more persistent than DDT. Fleming and coworkers [19] found that DDT and its metabolites were more likely to be present in brain tissue of Parkinson's disease (PD) patients than of control subjects. Also, Richardson et al. [17] reported significantly elevated levels of *pp*′-DDE in patients with PD versus controls. *pp*′-DDE induces neural death by apoptosis through the activation of mitogen-activated protein kinases (MAPKAs) [26]. 

Further we also tried to see the detected levels of OCP in both subjects of PD and control and found that one pesticide was higher in controls as compared to the PD subjects, which infers that there are other factors like gene predisposition that are contributing to the risk of PD. Also when the OCPs were subdivided into <5 ng/mL to >10 ng/mL groups, we found that *β*-HCH levels were higher than the other two pesticides in PD subjects when the levels were in the range of <5 ng/mL, suggesting that this OCP is strongly associated with PD risk. 

## 6. Conclusions 

The result obtained from this study clearly indicates that increased serum level of *β*-HCH and Dieldrin may be associated with the development of PD. Our results support the epidemiology-based studies that associate exposure to pesticides with increased risk of PD. This study also reflects that apart from the environmental factors there are other factors such as genetic that decide the disease development in susceptible individuals. 

## Figures and Tables

**Table 1:** Presence of organochlorine pesticides (OCPs) in Parkinson's disease (PD) and control subjects.

OCPs	Controls (*n* = 75)					Parkinson's disease (*n* = 70)				
	Detected in controls (%)	Detectable range in ng/mL	25%	50%	75%	Detected in Patients (%)	Detectable range in ng/mL	25%	50%	75%
*β*-HCH	13 (17.33)	0.67–2.7	1.0	1.42	1.75	37 (52.86)*	1.5–23.6	2.97	4.29	7.18
Dieldrin	7 (9.33)	0.96–4.2	0.98	1.1	2.04	43 (61.43)*	2.1–28.63	4.91	9.41	17.24
*pp*′-DDE	17 (22.66)	0.67–10.6	1.2	3.75	4.71	28 (40.0)*	1.4–12.5	3.59	4.35	6.5
*α*-HCH	3 (4.0)	1.3–1.45	1.37	1.45	1.45	16 (22.8)	1.3–12.6	1.99	3.11	3.78
*γ*-HCH	4 (5.33)	0.42–2.69	0.93	1.1	1.5	8 (11.4)	0.9–4.8	1.45	1.82	3.83
Aldrin	4 (5.33)	1.23–2.19	1.23	1.71	2.19	8 (11.4)	1.4–6.8	1.8	3.45	4.72
*α*-endosulfan	5 (6.67)	0.62–1.99	0.62	0.98	1.2	7 (10.0)	0.9–4.2	1.2	1.49	2.0
*β*-endosulfan	3 (4.0)	0.12–1.7	0.91	1.7	1.7	4 (5.7)	1.6–4.2	1.6	2.25	3.23
*pp*′-DDT	5 (6.67)	0.99–3.8	0.99	1.4	3.8	11 (15.7)	1.2–10.0	1.65	4.0	6.83

*OCPs which are present in ≥40% subjects of PD patients.

**Table 2:** Distribution and comparison of organochlorine pesticides, present in ≥40% subjects of Parkinson's disease.

Variables	Median		*U*-value	*z*-value	*r*-value	*P*-value
	Control	Case				
*β*-HCH	0	2.03	1467.000	−5.404	−0.448	0.000*
Dieldrin	0	4.20	1116.000	−7.043	−0.584	0.000*
*pp*′-DDE	0	0	2076.000	−2.650	−0.220	0.008*

*Significant *P* < 0.05 by Mann Whitney tests.

**Table 3:** Result of logistic regression.

	Collinearity statistics*		Variables in the equation^†^							
	Tolerance	VIF	*B*	S.E.	Wald	df	Sig.	OR	95% CI for OR	
									Lower	Upper
Step 1										
Age	0.92	1.08	−0.07	0.04	3.46	1	0.063	0.92	0.85	1.0
Sex	0.94	1.05	−0.61	0.57	1.14	1	0.285	0.54	0.17	1.67
*β*-HCH	0.83	1.20	0.94	0.21	19.17	1	0.000	2.56	1.68	3.91
Dieldrin	0.93	1.06	0.73	0.20	13.26	1	0.000	2.09	1.41	3.11
*pp*′-DDE	0.85	1.17	0.19	0.12	2.36	1	0.124	1.22	0.95	1.57
Constant	—	—	2.62	2.37	1.22	1	0.269	13.74	—	—

*Dependent variable: groups.

^†^Variable(s) entered on step 1: age, sex, *β*-HCH, dieldrin, *pp*′-DDE.
